# Pathology of Fever, &c. Founded on the Blood's Vitality

**Published:** 1825-04

**Authors:** John Jones


### Art. IV.—*Pathology of Fever, &c. founded on the Blood's Vitality.* By John Jones, Esq.

[Continued from page 115.]

From the preceding observations, morbid action appears greatly modified by three different states of the circulation, which are accompanied with corresponding changes in the functions of the nervous system:—1, venous congestion; 2, increased arterial action; 3, arterial action accompanying debility, constituting morbid irritability. Under these heads we proceed to consider some of the diseases, most illustrative of the foregoing pathological views.

1. *Diseases of congestion.*—These comprise a numerous class, both chronic and acute. The following, briefly considered, are sufficient for our present purpose:—1, idiopathic fever; 2, gout; 3, sanguineous apoplexy; 4, varicose veins.

1. *Idiopathic fever.*—The production of fever as an effect of venous congestion, conjoined with debilitating agents acting directly on the nervous system, has already been explained. If the depressing powers are great, they impede the development of reaction, render the system incapable of contending with the disease, and produce the various degrees of virulence so prevalent in fevers of hot climates, and occasionally occurring in those of our own. Plague, yellow fever, bilious remittent, slow, nervous, and putrid malignant fevers of Huxham, typhus, synochus, and intermittent, may all be considered as similarly produced, and acquiring their peculiar character from particular circumstances connected with their production. Thus plague, like the small-pox, generates a matter by which it is propagated, although greatly under the influence of a peculiar state of the atmosphere; and, therefore, spreading most widely in large and crowded cities. The yellow fever, bilious remittents of tropical climates, and intermittents of our own, are generated by putrid effluvia or marsh miasmata; and the simple continued fever, as it most frequently occurs with us, arises from a vitiated state of the atmosphere, acting on a constitution already predisposed to become feverish. The late Dr. Clark, in his work on Diseases which prevail in long Voyages to hot Climates, observes, "After many years' careful attention to the symptoms and nature of fever as they have occurred in practice, and after reading many authors on the subject, I am thoroughly convinced that, although many varieties happen, according to difference of constitution, season, situation, and climate, yet in many parts of the world the disease is essentially the same; or, in other words, consists of one genus; and that the only species that can be ascertained, are intermittents, remittents, and continued."

Three remarkable changes occur in fever, which is therefore properly considered as consisting of three stages.

*First stage.*—The proximate cause has been shown to consist in depressing agents acting on the nervous system, either directly or through the medium of the blood. We accordingly find the primary symptoms strongly indicative of diminished power. The patient complains of lassitude and disinclination to exertion, together with a general feeling of indisposition, and sensation of cold accompanied with chills. The head is affected with pain, vertigo, and tinnitus aurium. Eyes lose their animated expression, and appear heavy and languid. Pulse weak and slow. Nausea, and sometimes vomiting, occur, accompanied with painful distention of the epigastrium, particularly when pressed upon. Bowels, for the most part, constipated. The larger muscles, especially of the loins, are affected with pain. The knees feel weak and tremulous, which, together with the vertigo, causes a staggering in the gait, as if from intoxication. The sensation of cold and shivering alternates with partial and irregular heats, the face becoming flushed, whilst the extremities are cold.

This state continues, with more or less severity, for an uncertain period, according to the particular character of the fever, the extent of the predisposing and exciting causes, and the peculiar constitution of the patient. It often goes off spontaneously, or by the assistance of evacuants. When this is not the case, the symptoms indicative of more perfect reaction supervene, which constitute

*The second stage.*—The skin now becomes hot and dry, particularly in children. Pulse, from having been weak and oppressed, is stronger, fuller, and quicker. Face flushed; eyes often bloodshot and impatient of light, have an unmeaning stare, or an expression of apprehension, accompanied with increased pain in the head. A tendency to delirium occurs, first observable on awaking from disturbed slumbers, but soon becomes more constant. Breathing quick. Tongue dry and furred, or of a bright red and chopped; sometimes smooth and glossy. Teeth surrounded with a dark-coloured sordes. Taste depraved. Bowels continue costive. Urine high coloured, and scanty. As the disease advances, the sensorial functions become more deranged, and less in a state of excitement. The incoherent wanderings, which may have continued with little intermission, are changed into a low, muttering delirium and stupor, from which, however, the patient might be roused to answer questions; which he does, for the most part, in monosyllables, and immediately relapses into the same state. Eyes have no expression of intelligence. Deafness, which sometimes exists from the commencement, is now increased. Pulse weak and quick. Tongue continues parched; is protruded with difficulty; tremulous when shown; and has a flat or concave surface. Alvine evacuations, throughout the disease, have an unnatural appearance; for the most part, dark brown or nearly black. Although evening exacerbations have hitherto been regular, the paroxysms are now more partial, and excited by the slightest stimulants. The face is, perhaps, affected with irregular flushes, whilst the extremities are cold.

This derangement of the sensorial functions, which so peculiarly characterises this stage of typhus, when unaccompanied with inflammation of the brain, is the effect of diminished excitement, doubtless connected with venous congestion of that important organ. To visceral congestions, also, is to be attributed that loss of tone in the system, which is so remarkable in all fevers of the typhoid type.

The above symptoms continue for an uncertain period, varying from one to six weeks or more, when such as are indicative of exhaustion occur, which constitute the

*Third stage.*—The chance of recovery is here greatly diminished. For the most part, it terminates fatally in a few days, and in some cases even in a few hours. The pulse is weak, rapid, and intermittent. Breathing short and laborious. Heat of surface much diminished; the extremities cold, although partial reactions still continue. The countenance possesses that peculiar character known by the term *facies Hippocratica*; involuntary stools; subsultus tendinum, hiccup, jactitation, picking the bed-clothes, waving the arms, sinking towards the foot of the bed, are the symptoms which prevail more or less, and are indicative of a fatal termination. At this period the intellect often becomes clearer, and the patient appears conscious of his awful situation; but it is a momentary blaze, which immediately precedes the darkness of dissolution.

*Modifications of fever.*—The above is the usual progress of typhous fever as it occurs with us, but modified by organic determinations of blood, peculiar constitution of the patient, and the different degrees in the exciting and predisposing causes.

*Accompanied with topical inflammation.*—From the intimate connexion existing between venous congestion and arterial excitement, inflammation is exceedingly apt to occur at any period of the disease. When occurring at the commencement, it might readily be mistaken for one of the phlegmasiæ; but the continuance of the febrile symptoms after those of the local affection have subsided, and the peculiar derangement of the sensorial functions characterising typhus, soon point out its real nature. The organs most liable to be thus affected, are the brain, lungs, liver, and intestines. The brain is susceptible of three different states of the circulation, accompanied with corresponding symptoms:—1, venous congestion; 2, arterial excitement, not amounting to positive inflammation; 3, actual inflammation, constituting phrenitis. The first is attended with depression of the vital energies, as above described. The second is characterised by watchfulness, and a tendency to talk incoherently, particularly during febrile exacerbations; but the delirious wanderings are not so boisterous as in actual inflammation. In the third, symptoms of phrenitis are strongly marked, and, if not allayed, soon terminate fatally, by producing disorganisation, &c.

The following case is illustrative of cerebral excitement, not to the extent of positive inflammation. A young woman had been affected with incoherency, accompanied with severe pain in the head, for a week previous to my seeing her. She had a remarkable staggering in her gait, with the usual symptoms of typhoid fever. Evening exacerbations were regular, but heat of the surface not much increased. Pulse varied from 110 to 120, weak and small. Tongue dry. Bowels constipated, unless moved by medicines, when the evacuations were dark coloured. The fever continued, with little intermission, for five weeks, during which delirium was constant, but greater by night. The symptoms resisted general and local bleeding, emetics, blisters, and almost constant purgation; and at last were resolved by the formation, on the scalp, of numerous phlegmonous tumors, about the size of a marble, which readily suppurated, and proved the commencement of convalescence.

Sometimes inflammatory action of particular organs occurs in typhoid fever, when the system is so much depressed by visceral congestions as to be unable to support febrile excitement, and the reactions are consequently imperfect. A disposition to inflammation also occasionally characterises the prevailing epidemic. I have known four individuals in one family affected with typhus, in each of whom severe pain in the bowels was a prominent symptom. In one of them, a lad about fourteen years old, the disease terminated fatally in the course of a few days. His symptoms at first appeared but trifling: pulse weak, and not much accelerated; temperature of the skin but little increased; tongue moist. Severe pain of the abdomen, increased upon pressure, soon supervened; which was, however, relieved by fomentations, leeching, blistering, and laxatives. After leaving him apparently better, I was surprised on my next visit to find him *in articulo mortis.* His friends informed me that the pain had returned, and had been exceedingly violent, accompanied with constant retching, for twelve hours; of which they had neglected to apprise me.

This appears a strongly-marked case, in which the energies of the system were so greatly depressed by visceral congestions as to prevent febrile reaction. But, as reaction is a necessary consequence of the sudden depression of nervous power, the arterial excitement occurred in the vicinity of the congested organs, producing inflammatory action.

*Accompanied with diarrhœa.*—Although a disposition to costiveness usually exists in fever, diarrhœa often becomes a prominent symptom, which, if not judiciously moderated, accelerates a fatal termination. In such cases, febrile excitement, or reaction, is extremely imperfect, and consequently exhaustion is more readily induced. The irritability of the intestines, on which diarrhœa seems to depend, is doubtless the effect of increased vascular action, occasioned either by partial reactions, accompanying congestions of the abdominal viscera, or acrid alvine secretions. Although it is generally believed that diarrhœa might arise from relaxation of the excretory vessels, without increased vascular action, I am inclined to think that, in fever at least, this is never the case; and we accordingly find those measures which tend to lessen sanguineous determinations to the abdominal viscera most efficacious in removing this troublesome symptom, particularly when conjoined with opiates which have a remarkable effect in allaying irritation, when not amounting to positive inflammation.

*Modus Operandi of some of the Remedies employed in the Treatment of Fever.*

In considering the proximate cause of fever as consisting in depression of the system, which continues more or less through its whole course, occasioned especially by visceral congestions, the plan of treatment now generally approved is greatly confirmed. The indications are—1, to obviate the causes of depression, whether arising from organic congestions, sedative agents acting directly on the nervous system, or, as is most frequently the case, these two causes combined; 2, to allay inordinate vascular action; 3, to subdue symptoms of organic inflammation; 4, to restore the tone of the system after febrile action has subsided. These indications are sufficiently fulfilled by the remedies usually employed; the *modus operandi* of some of which we proceed to consider, in connexion with the preceding views.

*Emetics* determine powerfully to the surface, and thus tend to restore the functions of the skin, and at the same time to lessen or remove visceral congestions, before fever is completely established. They are, therefore, most beneficial when administered at the commencement of the disease, and often cut it short.

*Bleeding*, as a remedy in fever, has been recommended from the earliest period. Celsus observes, "Ergo vehemens febris, ubi rubit corpus, plenaque venæ tument, sanguinis detractionem requirit?" Our own acute practical physician, Sydenham, also strongly advises the free use of the lancet, even in plague. From the abuse of this excellent remedy, and likewise from erroneous pathological views, it has, for the last century, lost the confidence formerly reposed in it. Its utility, however, under certain limitations, is now beginning to be generally acknowledged. Like other depletory measures, it tends to obviate visceral congestions, and thus to remove the principal cause of the disease. It is more particularly useful in lessening the irritation of the system, and equalising the circulation during febrile excitement or reaction; and, when this state is well marked, should be rarely omitted. Although depletion, if too freely employed, might unnecessarily expend the vital energies, and accelerate a fatal termination, experience fully proves our apprehensions from this source to have been too great, and that we have verified the ancient adage—"Incidit in Scillam, cupidus vitare Charybdim."

*Purgatives.*—Till of late purgatives have always been used with great caution during typhoid fever, from the fear of increasing debility. Since Dr. J. Hamilton's valuable publication, however, their use has become more general, and they are now considered the practitioner's chief dependence. They lessen determinations of blood to the head; prevent pending congestions, either of the brain or abdominal viscera; and tend to remove them, if already existing. These salutary effects, which as evacuants alone they might produce, are more certainly insured by mercurial purgatives, which have a peculiar influence on the liver and the venous circulation in general.

In administering purgatives, particularly calomel, our chief object is to remove existing congestions. If given at the commencement of fever, they frequently cut it short, or, at least, tend to ameliorate subsequent symptoms. At this period calomel may be given in large and repeated doses, conjoined with other aperients, so as to insure copious and frequent evacuations. It cannot, however, be too frequently repeated, that active depletory measures are more particularly applicable to those forms of fever in which increased vascular action is strongly marked. In such cases we generally find that, in proportion to the freedom with which they have been employed, do the energies of the constitution improve. But every day's delay after the stage of excitement has commenced, renders such treatment certainly less useful, and proportionably hazardous, lest we accelerate, instead of avert, exhaustion of the vital powers.

*Mercury.*—The mercurial treatment of fever was first generally adopted in the West Indies by Dr. Chisholm, in 1794, although occasionally by others before that time. Mercury, given so as gently to affect the constitution, is peculiarly applicable to that form of fever in which reaction is feebly developed, as it tends to diminish organic congestions, without adding to the depression of the system. In such cases, when the gums begin to be affected, the febrile symptoms usually subside, and convalescence commences. These salutary effects appear to be produced by the powerful influence of the mercury on the venous circulation, by which the congested organs are restored to the due performance of their functions.

[To be continued.]

